# *Pasteurella multocida* activates apoptosis via the FAK-AKT-FOXO1 axis to cause pulmonary integrity loss, bacteremia, and eventually a cytokine storm

**DOI:** 10.1186/s13567-024-01298-7

**Published:** 2024-04-08

**Authors:** Guangfu Zhao, Yunhan Tang, Ruitong Dan, Muhan Xie, Tianci Zhang, Pan Li, Fang He, Nengzhang Li, Yuanyi Peng

**Affiliations:** 1 College of Veterinary Medicine, Southwest University, Chongqing, China; 2 Department of Environment and Safety Engineering, Taiyuan Institute of Technology, Taiyuan, China; 3 Department of Endocrinology and Metabolism, Center for Diabetes and Metabolism Research, West China Hospital, Sichuan University, Chengdu, China

**Keywords:** *Pasteurella multocida*, apoptosis, pathogenesis, FAK-AKT-FOXO1 axis, pneumonia, pulmonary integrity, cytokine storm

## Abstract

*Pasteurella multocida* is an important zoonotic respiratory pathogen capable of infecting a diverse range of hosts, including humans, farm animals, and wild animals. However, the precise mechanisms by which *P. multocida* compromises the pulmonary integrity of mammals and subsequently induces systemic infection remain largely unexplored. In this study, based on mouse and rabbit models, we found that *P. multocida* causes not only lung damage but also bacteremia due to the loss of lung integrity. Furthermore, we demonstrated that bacteremia is an important aspect of *P. multocida* pathogenesis, as evidenced by the observed multiorgan damage and systemic inflammation, and ultimately found that this systemic infection leads to a cytokine storm that can be mitigated by IL-6-neutralizing antibodies. As a result, we divided the pathogenesis of *P. multocida* into two phases: the pulmonary infection phase and the systemic infection phase. Based on unbiased RNA-seq data, we discovered that *P. multocida*-induced apoptosis leads to the loss of pulmonary epithelial integrity. These findings have been validated in both TC-1 murine lung epithelial cells and the lungs of model mice. Conversely, the administration of Ac-DEVD-CHO, an apoptosis inhibitor, effectively restored pulmonary epithelial integrity, significantly mitigated lung damage, inhibited bacteremia, attenuated the cytokine storm, and reduced mortality in mouse models. At the molecular level, we demonstrated that the FAK-AKT-FOXO1 axis is involved in *P. multocida*-induced lung epithelial cell apoptosis in both cells and animals. Thus, our research provides crucial information with regard to the pathogenesis of *P. multocida* as well as potential treatment options for this and other respiratory bacterial diseases.

**Supplementary Information:**

The online version contains supplementary material available at 10.1186/s13567-024-01298-7.

## Introduction

Since the COVID-19 outbreak, people worldwide have come to realize that respiratory pathogens can cause a public health and economic crisis. In fact, respiratory infections affect approximately 4 million people worldwide annually [1]. In animal husbandry, animals also suffer from respiratory infections, such as avian influenza (AI), bovine respiratory disease complex (BRDC), and porcine respiratory disease complex (PRDC), which cause billions of dollars of losses to the agricultural industry and disrupt food supply chains [2–4]. To guarantee oxygen exchange and provide protection from pathogen invasion, the integrity of the pulmonary epithelium is crucial. When the pulmonary epithelium becomes compromised, respiratory pathogens have a greater chance of invading the body and causing more severe diseases. Some respiratory pathogens actually aim to compromise the integrity of the host pulmonary epithelium to spread their infections to extrapulmonary tissues, which not only enables the bacteria to survive but also transforms a local infection into a systemic infection [5–7].

Apoptosis, a type of programmed death, is essential for maintaining physiological processes in all living organisms [8]. Uncontrolled cell death threatens tissue integrity, and to avoid unexpected apoptosis, cells have evolved a set of antiapoptotic mechanisms. Programmed cell death contributes to organismal homeostasis when the body faces nonvirulent encounters, but its role in infection is complex and pathogen-dependent [9, 10]. Apoptosis is known to play an indispensable role in the pathophysiology of a variety of diseases, such as inflammatory bowel disease (IBD), enteropathogenic and enterohemorrhagic *Escherichia coli*-induced enteritis and acute lung injury [11–13]. However, more studies are needed to determine whether bacterium-induced pulmonary apoptosis has an impact on the loss of pulmonary epithelial integrity and the development of systemic infection.

*Pasteurella multocida* is a gram-negative zoonotic pathogen that is known for inducing respiratory diseases in a variety of animals, including farm animals (cattle, rabbit, pig and chicken), wild mammals, reptiles, and laboratory animals [14]. Recent clinical studies have shown that *P. multocida* is known to cause human respiratory problems and consequent mortality [15]. *P. multocida* can be categorized into five groups (A, B, D, E and F) based on the characteristics of the bacterial capsular polysaccharide [16]. Among them, capsular types A, D and F are notorious for causing respiratory diseases in both animals and humans [17]. As a respiratory pathogen, *P. multocida* infection, particularly capsular type A infection, frequently causes clinically detectable pulmonary damage [18]. Nevertheless, there is a growing body of evidence indicating that *P. multocida*, including capsular type A, can both damage the host's respiratory system and leak into its blood circulation system (bacteremia), leading to extrapulmonary infections [19–21]. Notably, *P. multocida* bacteremia and *P. multocida*-induced extrapulmonary damage have become increasingly common in the clinic, suggesting that *P. multocida*-induced systemic infection could be an important part of its pathogenesis [22–24]. As a result, we hypothesized that a complete *P. multocida* infection model could comprise at least two phases: a pulmonary infection phase (Phase 1) and a systemic infection phase (Phase 2).

In the present study, we tested our two-phase hypothesis, investigated the role of bacteremia during *P. multocida* infection, and explored the molecular mechanism by which *P. multocida* compromises the integrity of the pulmonary epithelium. Our results demonstrated that bacteremia caused by the loss of pulmonary epithelial integrity greatly contributed to *P. multocida* pathogenesis, as evidenced by the induction of a cytokine storm. At the molecular level, *P. multocida* can manipulate pulmonary epithelial cell apoptosis by altering the FAK-AKT-FOXO1 axis, causing a loss of pulmonary epithelial integrity. Conversely, selectively inhibiting apoptosis can significantly mitigate *P. multocida* infection.

## Materials and methods

### Bacterial strains and culture conditions

*Pasteurella multocida* PmCQ2 (serotype A, GenBank accession no. CP033599) was isolated from bovine pneumonia in Chongqing, China, with a one-week LD_50_ of 1 CFU. *P. multocida* PmCQ6 (serotype A, GenBank accession no. CP033600) was isolated from the upper respiratory tract of cattle in Chongqing, China, and was 10^8^ times less virulent than PmCQ2. All *P. multocida* strains were cultured on Martin’s broth agar and 5% horse serum at 37 °C.

### Animal experiment

The animal experiments followed the National Research Council's “Guide for the Care and Use of Laboratory Animals” (8th edition). The animal protocols used were approved by the Institutional Animal Care and Use Committee (IACUC), Southwest University, China (No. LAC2023-1-0232). Eight-week-old male CD-1 (ICR) mice weighing 30–35 g were purchased from HFK Bioscience (Beijing, China). Two-month-old male New Zealand rabbits weighing 1500 g were purchased from Enswell Biotechnology (Chongqing, China). Animals were housed in individual IVC cages with a 12 h/day light/dark cycle, a temperature maintained at 25 °C, and free access to food and water. Animals were randomly assigned to each experimental group.

For infection experiments, animals were anaesthetized with 1.5% pentobarbital sodium and then intranasally infected with 10^4^ (mice) or 10^8^ (rabbits) CFU PmCQ2 or 10^7–8^ CFU PmCQ6. Ac-DEVD-CHO (CHO, CSNpharm, CSN23216), Evens Blue (Solarbio, IE0280), an anti-mouse IL-6-InVivo antibody (Selleck, A2118), a rat IgG1 isotype control-InVivo (Selleck, A2119) and saline were injected into the mice according to the schematic presentations (in the Results section). After finishing the animal experiments, the mice were anaesthetized with 1.5% pentobarbital sodium and then euthanized for blood and tissue collection. For the Evans blue assay, lung solutions and serum were measured for optical density at 620 nm (OD_620_). Changes in pulmonary permeability = OD_620_ of the lung solution/OD_620_ of the corresponding serum.

### Histological and immunohistochemical examinations

The collected tissues were immediately fixed with 4% paraformaldehyde (w/v), embedded in paraffin and sectioned at 5 µm thickness. Haematoxylin and eosin (H&E) staining (Beyotime Biotechnology, China), Masson’s trichrome staining (Baso, China), and periodic acid Schiff (PAS) staining (Solarbio, China) were performed according to the manufacturer’s instructions. Briefly, for immunohistochemical examinations, the slides were deparaffinized, rehydrated and retrieved. Then, the slides were treated with 3% H_2_O_2_ and blocked with 5% bovine serum albumin. Slides were incubated with primary antibodies against p-AKT (Proteintech, 66444-1-Ig) and ITGAV (HUABIO, ET1610-15), followed by incubation with the corresponding secondary antibodies conjugated to horseradish peroxidase (HRP) (Zsbio, PV-6000). Finally, a DAB kit (Zsbio, ZLI-9018) was used to visualize the slides, which were counterstained with haematoxylin.

### Immunofluorescence

The slides were deparaffinized, rehydrated and retrieved. Then, the slides were blocked with 5% bovine serum albumin, followed by incubation with primary antibodies against SPC (Proteintech, 10774-1-AP) or NGAL (ABclonal, A2092). Primary antibodies were added on the second day, and the slides were incubated with Cy3-labelled secondary antibodies (ABclonal, AS007). Finally, the slides were counterstained with DAPI and visualized by confocal fluorescence microscopy.

### Cell treatment

The mouse-immortalized lung epithelium cell line TC-1 was maintained at 37 °C in a 5% CO_2_ atmosphere. The cells were cultured in Dulbecco’s modified Eagle’s medium (DMEM, HyClone) supplemented with 10% foetal bovine serum, penicillin (100 U/mL) and streptomycin (100 mg/mL). For *P. multocida* infection, cells were infected with log-phase PmCQ2 at an MOI of 1.

For the apoptosis Annexin V-PI staining tests, the harvested cells were stained with Annexin V-FITC and PI according to the manufacturer’s instructions for the Annexin V-FITC/PI Apoptosis Kit (Elabscience, E-CK-A211). Finally, apoptotic cells were quantified by flow cytometry.

For Transwell assays, 8.0 µm pore-size Transwell inserts (Corning, 3422) were used. Briefly, antibiotic-free medium with or without drugs was added to both the upper and bottom chambers, and 1 × 10^5^ cells were seeded in the upper chambers for 16 h. Then, PmCQ2 at an MOI of 1 was added to the upper chambers to incubate with the cells. After 8 h of incubation, the bottom and upper media were collected and diluted to an appropriate concentration to measure the number of translocated bacteria via the plate counting method. Translocation rate = number of translocated bacteria/(number of upper bacteria + number of translocated bacteria) × 100%.

### In situ apoptosis staining (TUNEL assays)

In situ TUNEL Staining was performed according to the manufacturer’s instructions for the One-step TUNEL In Situ Apoptosis Kit (Elabscience, E-CK-A320). Briefly, dewaxed slides were treated with proteinase K and incubated with TdT enzyme in TdT equilibration buffer, followed by labelling with labelling solution (FITC). Finally, the slides were counterstained with DAPI and visualized by fluorescence microscopy.

### Quantitative real-time PCR

Total RNA was extracted with an AFTSpin Tissue/Cell Fast RNA Extraction Kit for Animals (ABclonal, RK30120) and reverse transcribed into a cDNA pool with ABScript III RT Master Mix for qPCR with gDNA Remover (ABclonal, RK20429). Real-time PCR was performed with a Bio-Rad CFX96 instrument. The relative mRNA expression was normalized to the expression of beta-actin mRNA. The complete real-time primer sequences are presented in Additional file 8.

### RNA sequencing

The RNA-sequencing service was provided by Personal Biotechnology Co., Ltd., Shanghai, China. Briefly, mouse lungs were homogenized in liquid nitrogen, after which total RNA was extracted using TRIzol reagent. rRNA was removed using the Ribo-Zero rRNA Removal Kit (Illumina, MRZH116). Each specimen made up a total of 1 μg of the cDNA library. An Agilent 2100 Bioanalyzer was then used to estimate the size of the cDNA library. Finally, 150 bp paired-end reads were produced from the cDNA sequence via the Illumina HiSeq 4000 platform. The original RNA-seq data are available in Additional file 1.

### Cell transfection and shRNA-mediated knockdown

shRNA knockdown DNA sequences were cloned and inserted into the pLKO.1 plasmid. Cells were transfected with 2 μg of the pLKO.1 plasmid via the Lipo3000 transfection agent (Thermo Fisher, L3000008) according to the manufacturer’s instructions. The sequence information is presented below.

shFOXO1-1: TGGAAACCAGCCAGCTATAAA;

shFOXO1-2: CCGCCAAACACCAGTCTAAAT.

### Western blotting

RIPA buffer containing protease inhibitor cocktail was used to lyse cells in culture wells. For tissues, a homogenization step was needed. The protein in the supernatant was obtained by centrifugation at 4 °C. The bicinchoninic acid method (Thermo Fisher, #23227) was used to quantify the protein concentration of the cell lysates, followed by boiling with a reducing agent (DTT). Protein samples were resolved via SDS‒PAGE, transferred to a PVDF membrane, blocked with 5% skim milk and incubated with the corresponding primary antibodies against Cleaved Caspase-3 (Cell Signaling Technology, #9664 and ImmunoWay, YC0006), Caspase 7 (Proteintech, 27155-1-AP), p-mTOR Ser2448 (Cell Signaling Technology, #5536), mTOR (Abways, CY5306), p-AKT Ser473 (Cell Signaling Technology, #4060), AKT (Cell Signaling Technology, #9272), p-FAK Tyr397 (Cell Signaling Technology, #3283), FAK (HUABIO, ET1602-25), FOXO1 (Cell Signaling Technology, #2880), p-FOXO1 Thr24 (Cell Signaling Technology, #2599), ITGAV (HUABIO, ET1610-15), BAX (Cell Signaling Technology, #2772), ACTIN (Proteintech, 81115-1-RR) and GAPDH (ABclonal, AC033). Finally, the PVDF membranes were incubated with the corresponding HRP-conjugated secondary antibodies (Boster Bio, BA1054, BA1050 and BA1060), followed by visualization with an enhanced chemiluminescence (ECL) kit (Bio-Rad, #170-5060). Western blot bands were normalized to those of beta-actin or GAPDH and quantified using ImageJ software.

### Enzyme-linked immunosorbent assay (ELISA)

Briefly, tissue samples were homogenized, frozen, thawed twice, and centrifuged. The supernatants were subjected to ELISA according to the manufacturer’s protocol for the Mouse ELISA Kit with Plates (88-7064-86 for IL-6, 88-7013-22 for IL-1β, and 88-7346-88 for TNF-α; Invitrogen).

### Statistical analysis

For the statistical analysis, GraphPad Prism (Prism 6.0) and PASW Statistical 18.0 (SPSS) software were used. To group sets of unique data, the mean and standard deviation (SD) were used. The significance of the differences in the data were determined using two-tailed Student’s *t* tests, Mann‒Whitney tests, or one-way ANOVA. A log-rank (Mantel‒Cox) test was used to measure the survival rates after a Kaplan‒Meier curve was generated. The statistical significance is represented as follows. *: *p* < 0.05, **: *p* < 0.01, ***: *p* < 0.001.

## Results

### *Pasteurella multocida* disrupts pulmonary epithelial integrity, resulting in bacteremia and extrapulmonary infections

**Figure 1:**
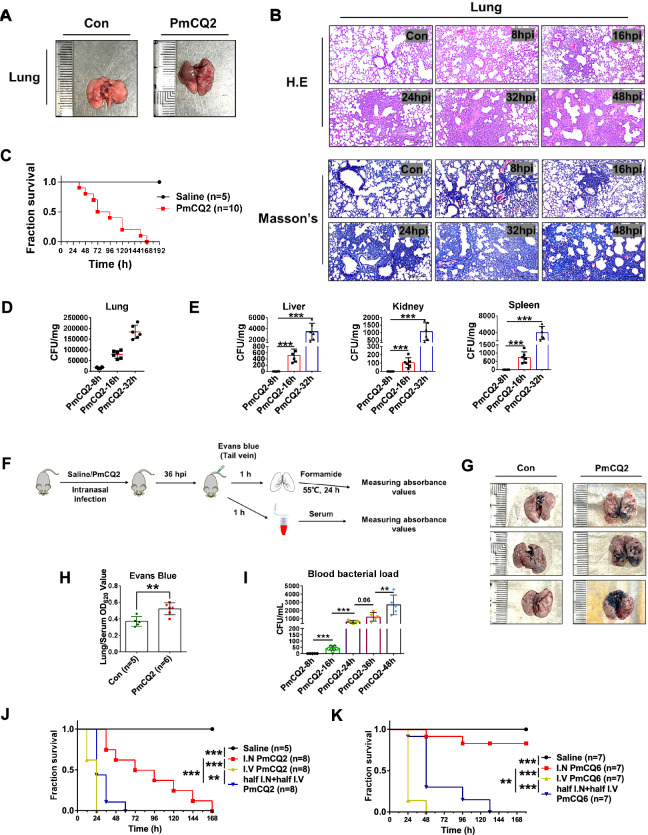
***Pasteurella multocida *infection destroys pulmonary epithelial integrity and induces lethal bacteremia**. **A** Photographs of murine lungs infected with or without 10^4 ^CFU of PmCQ2. **B** Representative images of H&E staining and Masson’s trichrome staining of murine lungs infected with 10^4 ^CFU of PmCQ2. Scale bars = 200 μm. **C** Survival curves of mice infected with 10^4^ CFU of PmCQ2. **D** The bacterial load of murine lungs infected with 10^4 ^CFU of PmCQ2. *N* = 6. The data are presented as the means ± SDs. **E** The bacterial load of murine extrapulmonary tissues (liver, kidney, and spleen) intranasally infected with 10^4 ^CFU of PmCQ2. *N* = 6. The data are presented as the means ± SDs. **F** A schematic of the Evans blue treatment protocol. **G** Representative photographs of murine lungs after Evans blue treatment. **H** Quantification of Evans blue in the lungs and serum obtained from PmCQ2-infected mice and control mice. *N* = 5. The data are presented as the means ± SDs. **I** Blood bacterial load of PmCQ2-infected mice at different time points. *N* = 5. The data are presented as the means ± SDs. **J** Survival curves of control group mice, intranasal infection group mice, intravenous infection group mice and half intranasal half intravenous infection group mice. All infection groups were infected with a total of 10^4 ^CFU PmCQ2, while the control group mice received saline. **K** Survival curves of control group mice, intranasal infection group mice, intravenous infection group mice and half intranasal half intravenous infection group mice. All infection groups were infected with a total of 10^8 ^CFU of PmCQ6, while the control group mice received saline. Every single point represents one individual. ** *p* < 0.01, *** *p* < 0.001.

In our previous work [21], we found that mice intranasally infected with PmCQ2 exhibited severe lung damage and extrapulmonary infections. Here, we repeated previous work and found that intranasal infection with 1 × 10^4^ CFU of lethal PmCQ2 indeed induced serious lung damage in mice in a time-dependent manner (Figures 1A through C). Consistent with our previous report, not only pulmonary infection but also extrapulmonary infections were confirmed (Figures 1D and E). In agreement with previous reports [25, 26], we confirmed that the proinflammatory cytokines IL-6, TNF-α, and IL-1β accumulated in the lungs of PmCQ2-infected mice (Additional file 2), which is a typical response to pneumonia. Notably, since PmCQ2 was not detected in extrapulmonary tissues until 16 h post-infection (hpi), we realized that extrapulmonary infections appeared at approximately 16 hpi when the lung tissues were obviously damaged. These results suggest that *P. multocida* infection can be divided into two phases according to the presence or absence of extrapulmonary infections.

We then intravenously injected the mice into the tail vein with 30 mg/kg Evans blue (Figure 1F), which is widely used to assess the permeability of the pulmonary epithelial-blood barrier [27, 28]. As shown in Figures 1G and H, mice infected with PmCQ2 exhibited significantly increased permeability of the pulmonary epithelium-blood barrier, indicating a loss of lung epithelial integrity. Accordingly, at 16 hpi, the blood bacterial load clearly increased and was positively correlated with the duration of *P. multocida* infection (Figure 1I). Similarly, we found that intranasal infection with PmCQ2 in rabbits also resulted in severe pulmonary injury and bacteremia (Additional file 3). Given that it takes approximately 16 h for *P. multocida* to induce bacteremia, we artificially advanced the time to bacteremia by intravenous *P. multocida* injection. *P. multocida* PmCQ6 was isolated from the upper respiratory tract of cattle and was found to be 10^8^ times less virulent than PmCQ2 [29]. Compared with intranasal infection, intravenous infection or half intranasal plus half intravenous infection with PmCQ2 or PmCQ6 significantly increased mortality in mice, suggesting that bacteremia is closely related to the pathogenesis of *P. multocida* (Figures 1J and K). Together, these results indicate that *P. multocida* infection destroys pulmonary epithelial integrity, subsequently induces bacteremia, and ultimately causes systemic infection.

### *Pasteurella multocida* bacteremia accelerates host death via cytokine storms

**Figure 2:**
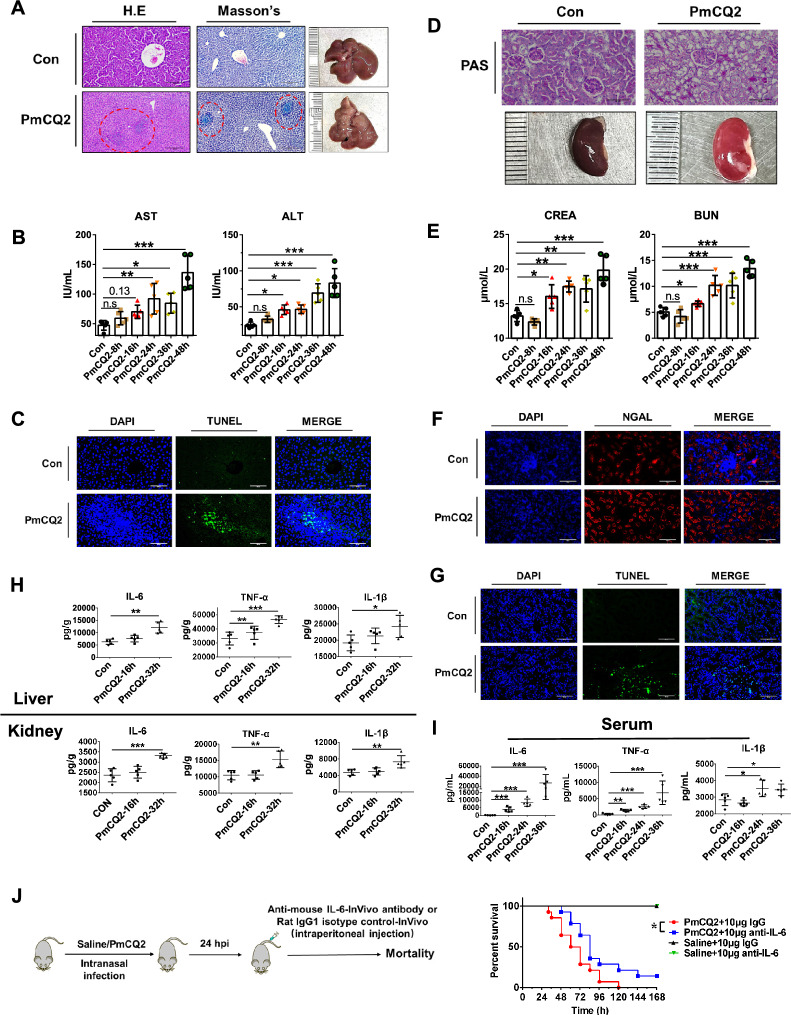
***Pasteurella multocida *bacteremia accelerates host death by inducing a cytokine storm**. **A** Representative images, H&E staining and Mason’s trichrome staining of PmCQ2-infected murine livers and control murine livers at 36 hpi. The obvious damage areas are indicated by red circles. Scale bar = 200 μm. **B** Quantification of aspartate aminotransferase (AST) and alanine aminotransferase (ALT) levels in PmCQ2-infected murine serum. Each group contained 5 replicates. *N* = 5. The data are presented as the means ± SDs. **C** Representative images of TUNEL staining of control murine livers and PmCQ2-infected murine livers at 36 hpi. The green puncta indicate dead cells. Scale bar = 200 μm. **D** Representative images of PAS staining of PmCQ2-infected murine kidneys and control murine kidneys at 36 hpi. Scale bar = 100 μm. **E** Quantification of plasma urea nitrogen (BUN) and serum creatinine (CREA) levels in PmCQ2-infected mice. Each group contained 5 replicates. *N* = 5. The data are presented as the means ± SDs. **F** Representative images of immunofluorescence analysis of kidney NGAL expression in PmCQ2-infected mice and control mice at 36 hpi. Scale bar = 100 μm. **G** Representative images of TUNEL staining of control murine livers and PmCQ2-infected murine kidneys. The green puncta indicate dead cells. Scale bar = 100 μm. **H** Quantification of IL-6, TNF-α, and IL-1β in the livers and kidneys of mice infected with or without PmCQ2 by ELISA at 24 hpi. *N* = 5. The data are presented as the means ± SDs. **I** Quantification of IL-6, TNF-α and IL-1β levels in the serum of mice infected with or without PmCQ2 by ELISA at 24 hpi. *N* = 5. The data are presented as the means ± SDs. **J** A schematic of the IL-6 in vivo antibody treatment protocol. On the right are survival curves of mice infected with or without PmCQ2 and treated with 10 μg of an in vivo IL-6 antibody or a rat IgG1 isotype. Each group was composed of 8 mice. Every single point represents one individual. * *p* < 0.05, ** *p* < 0.01, *** *p* < 0.001.

Next, we investigated the mechanism by which *P. multocida* bacteremia promotes host death. We examined the extrapulmonary tissues (liver and kidney) after *P. multocida* infection at the tissue and biochemical levels. As shown in Figures 2A and B, H, and E, Masson’s trichrome staining and biochemical tests suggested that *P. multocida* induced obvious liver injury and significantly increased the serum aspartate aminotransferase (AST) and alanine amino transferase (ALT) levels. TUNEL staining also showed that *P. multocida* infection increased the death of hepatocytes (Figure 2C). Similarly, PAS staining and biochemical tests also demonstrated *P. multocida*-induced kidney damage, as indicated by a reduction in the brush border of renal tubules and significant increase in plasma creatinine (CREA) and urea nitrogen (BUN) levels (Figures 2D and E). Correspondingly, immunofluorescence and TUNEL staining also showed that *P. multocida* induced the accumulation of neutrophil gelatinase–associated lipocalin (NGAL) and renal cell death (Figures 2F and G), and NGAL is a marker of acute kidney injury [30]. In addition, we found that intranasal infection with PmCQ2 in rabbits also resulted in severe liver injury (Additional file 4). These results indicated that *P. multocida* bacteremia leads to multiple extrapulmonary organ damage. Moreover, we determined that the levels of the proinflammatory cytokines IL-6, TNF-α and IL-1β in the liver, kidney and serum (systemic) were markedly increased (Figures 2H and I), suggesting that *P. multocida* bacteremia induces massive proinflammatory factor release. Considering that multiple-organ damage and massive proinflammatory cytokine release are typical phenotypes of cytokine storms [31], IL-6-neutralizing antibodies were used for intervention. As shown in Figure 2J, mice that received IL-6 neutralizing antibodies had significantly prolonged survival time and increased survival rates, suggesting that *P. multocida* bacteremia causes host death by inducing a cytokine storm. Together, the above results suggest that *P. multocida* not only infects the lungs but can also infect extrapulmonary tissues through bacteremia and ultimately lead to a cytokine storm.

### *Pasteurella multocida*-induced pulmonary epithelial cell apoptosis contributes to the loss of pulmonary epithelial integrity

**Figure 3:**
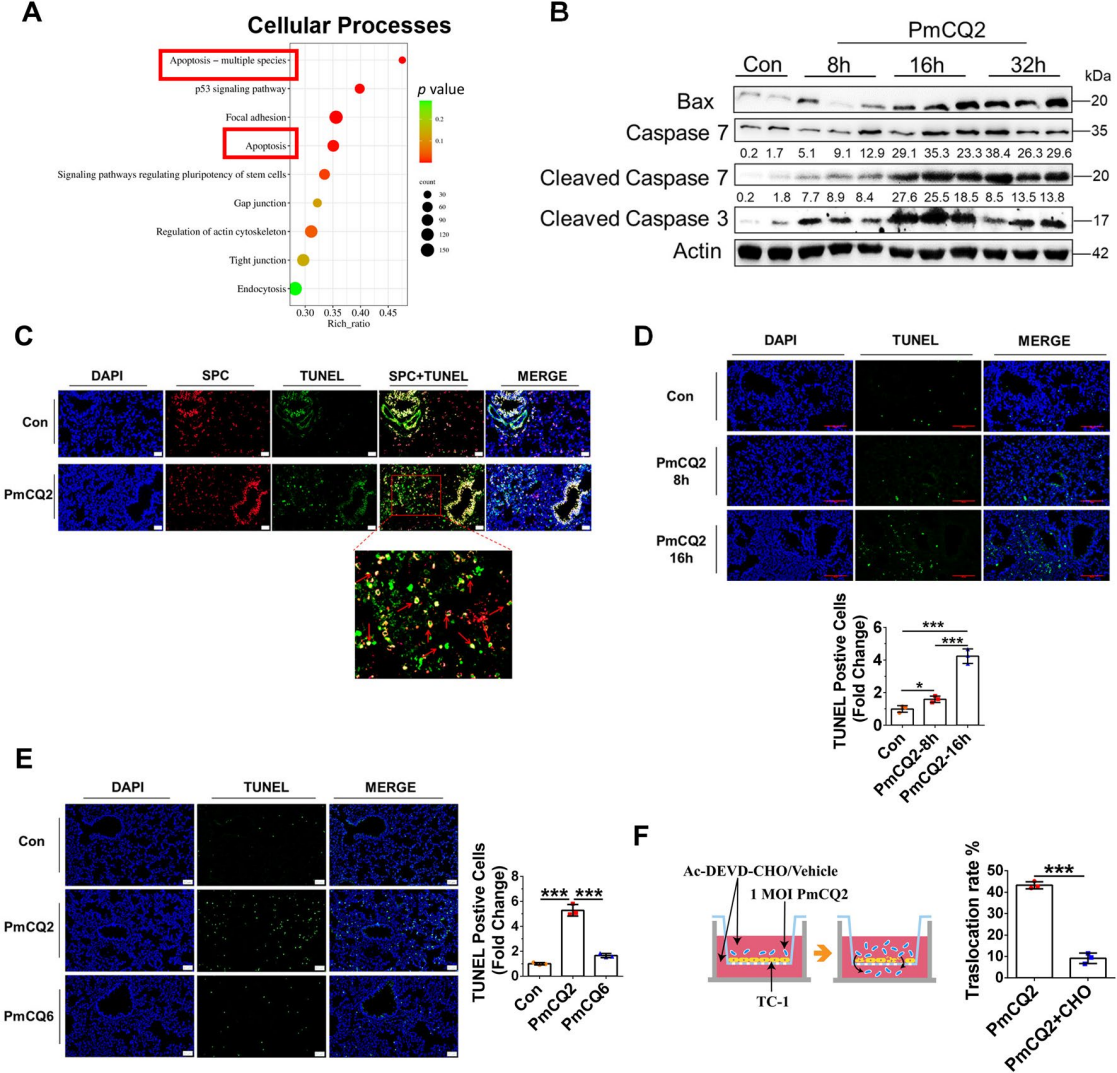
***Pasteurella multocida-*induced pulmonary epithelial cell apoptosis contributes to the loss of pulmonary epithelial integrity**. **A** Top ten RNA-seq enrichment pathways of cellular processes in murine lungs infected with 10^4^ CFU of PmCQ2 at 40 hpi. **B** Western blot analysis of murine lungs infected with 10^4 ^CFU of PmCQ2 at different time points. Each lane represents one individual. **C** Representative images of confocal analysis of SPC staining and TUNEL staining in in situ murine lungs infected with 10^4 ^CFU of PmCQ2 or saline at 16 hpi. Scale bar = 20 μm. The red arrow indicates an apoptotic lung epithelial cell. **D** Representative images of TUNEL staining of murine lungs infected with 10^4^ CFU of PmCQ2 at different time points. Scale bar = 200 μm. At the bottom are quantifications of TUNEL-positive cells in 3 random sections. The data are presented as the means ± SDs. **E** Representative images of TUNEL staining of murine lungs infected with 10^4^ CFU of PmCQ2 or 10^7 ^CFU of PmCQ6 at 24 hpi. Scale bar = 50 μm. On the right, quantification of TUNEL-positive cells in 3 random sections is shown. The data are presented as the means ± SDs. **F** A schematic of the Transwell assay protocol. On the right, quantification of the number of translocated bacteria in TC-1 cells treated with 20 μM of the apoptosis inhibitor Ac-DEVD-CHO or saline at 8 hpi. Each group contained 3 replicates. The data are presented as the means ± SDs. * *p* < 0.05, *** *p* < 0.001.

To explore the underlying mechanism by which *P. multocida* disrupts pulmonary epithelial integrity, we performed RNA-seq of lung tissues at 40 hpi. Through bioinformatics analysis, we found that the apoptosis pathway was one of the top 10 enriched pathways in the RNA-seq data (Figure 3A). Next, we demonstrated that the pro-apoptosis protein Bax and the apoptosis executioners Cleaved Caspase-3, and Cleaved Caspase-7 were significantly upregulated in murine lungs during *P. multocida* infection (Figure 3B). Correspondingly, in situ apoptotic pulmonary epithelial cells were confirmed in murine models by confocal microscopy, which showed positivity for the pulmonary epithelial marker SPC and TUNEL staining (Figure 3C) [32]. In addition, TUNEL staining revealed a large number of apoptotic cells in the PmCQ2-infected rabbit model (Additional file 5). In our murine models, the number of in situ apoptotic cells was significantly greater at 16 hpi than at 8 hpi, which is consistent with the development of bacteremia (Figure 3D). Moreover, fatal PmCQ2 induced many more apoptotic cells than nonfatal PmCQ6 in vivo (Figure 3E), suggesting that pulmonary apoptosis is tightly associated with the pathogenesis of *P. multocida*. Next, we tested whether *P. multocida*-induced apoptosis contributed to the loss of pulmonary epithelial integrity based on in vitro Transwell models. Ac-DEVD-CHO is a biologically active validated Caspase-3 inhibitor with the PARP cleavage site DEVD, and it has a low nanomolar Ki for Caspase-3 and Caspase-7. As shown in Figure 3F, compared with those treated with vehicle, cells treated with the apoptosis inhibitor Ac-DEVD-CHO (CHO) exhibited significantly reduced PmCQ2 translocation, suggesting that *P. multocida*-induced apoptosis enhances pulmonary epithelial integrity. Taken together, these results indicated that *P. multocida*-induced pulmonary epithelial cell apoptosis contributes to the loss of pulmonary epithelial integrity.

### Pharmaceutically blocking pulmonary epithelial cell apoptosis attenuates *Pasteurella multocida* infection

**Figure 4:**
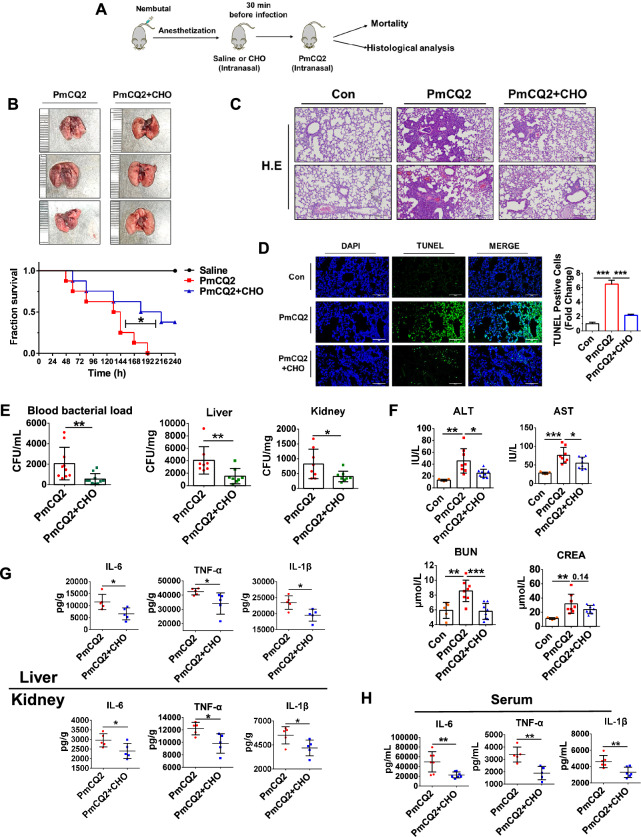
**Pharmaceutically blocking pulmonary epithelial cell apoptosis attenuates *Pasteurella multocida * infection**. **A** A scheme showing the Ac-DEVD-CHO (CHO) treatment protocol. **B** Representative images of PmCQ2-infected murine lungs with or without CHO treatment. At the bottom are survival curves of PmCQ2-infected mice with or without CHO treatment. Each group was composed of 8 mice. **C** Representative images of H&E staining of PmCQ2-infected murine lungs with or without CHO treatment at 24 hpi. Scale bar = 200 μm. **D** Representative images of TUNEL staining of PmCQ2-infected murine lungs with or without CHO treatment at 24 hpi. Scale bar = 200 μm. On the right, quantification of TUNEL-positive cells in 3 random sections is shown. The data are presented as the means ± SDs. **E** Bacterial load in the blood, liver, and kidney of PmCQ2-infected mice with or without CHO treatment. *N* = 8. The data are presented as the means ± SDs. **F** Quantification of plasma BUN and creatinine (CREA) and serum AST and ALT levels in PmCQ2-infected mice with or without CHO treatment at 24 hpi. N (con) = 5, *N* (PmCQ2) = 8, *N* (PmCQ2 + CHO) = 8. The data are presented as the means ± SDs. **G** Quantification of IL-6, TNF-α, and IL-1β levels in the liver and kidney of PmCQ2-infected mice with or without CHO treatment at 24 hpi by ELISA. *N* = 5. The data are presented as the means ± SDs. **H** Quantification of IL-6, TNF-α, and IL-1β levels in the serum of PmCQ2-infected mice with or without CHO treatment at 24 hpi by ELISA. Every single point represents one individual. * *p* < 0.05, ** *p* < 0.01, *** *p* < 0.001. *N* (IL-6) = 7, *N* (TNF-α) = 5, *N* (IL-1β) = 6. The data are presented as the means ± SDs.

To further examine whether lung epithelial apoptosis contributes to the pathogenesis of *P. multocida*, we administered 3 mg/kg of the apoptosis inhibitor CHO to mice intranasally infected with PmCQ2 (Figure 4A). As shown in Figures 4B through D, CHO treatment significantly reduced lung damage and the number of apoptotic cells, as evidenced by histological and in situ TUNEL analyses. Importantly, mice that received CHO treatment exhibited markedly prolonged survival and increased survival (Figure 4B), indicating that pulmonary epithelial cell apoptosis contributes to the pathogenesis of *P. multocida*. CHO treatment also significantly reduced the bacterial load in blood and extrapulmonary tissues (Figure 4E), suggesting that CHO partly rescues the loss of pulmonary epithelial integrity. Correspondingly, the *P. multocida*-induced cytokine storm was also partly rescued, as indicated by decreases in AST, ALT, CREA and BUN levels and reductions in IL-6, TNF-α and IL-1β levels in the liver, kidney and serum (Figures 4F through H). The above findings indicate that pulmonary epithelial cell apoptosis contributes to *P. multocida* pathogenesis and that pharmaceutically blocking pulmonary epithelial cell apoptosis can be a novel strategy against *P. multocida* infection.

### *Pasteurella multocida* activates pulmonary epithelial cell apoptosis via the FAK-AKT-FOXO1 axis

**Figure 5:**
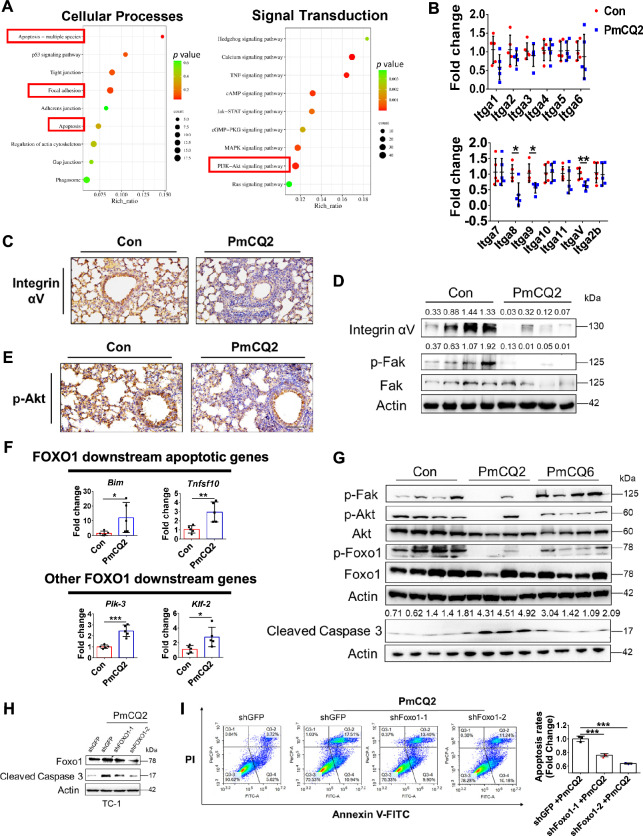
***Pasteurella multocida *activates pulmonary epithelial cell apoptosis via the FAK-AKT-FOXO1 axis**. **A** Top ten RNA-seq enrichment pathways of cellular processes and signal transduction in murine lungs infected with 10^4^ CFU of PmCQ2 at 16 hpi. **B** Transcription of the integrin α family in the lungs of PmCQ2-infected mice, as determined by qPCR. *N* = 5. The data are presented as the means ± SDs. **C** Representative images of integrin αV expression in the in situ lungs of PmCQ2-infected mice at 16 hpi, as determined by immunohistochemistry. Scale bar = 100 μm. **D** Western blot analysis of integrin αV, p-Fak and Fak in the lungs of PmCQ2-infected mice. Each lane represents one individual. **E** Representative images of the expression of p-Akt in the in situ lungs of PmCQ2-infected mice at 16 hpi, as determined by immunohistochemistry. Scale bar = 100 μm. **F** Transcription of Foxo1 downstream genes in the lungs of PmCQ2-infected mice at 16 hpi, as determined by qPCR. Each group contained 5 replicates. The data are presented as the means ± SDs. **G** Western blot analysis of the FAK-AKT-FOXO1 pathway in the lungs of PmCQ2-infected mice and PmCQ6-infected mice at 16 hpi. Each lane represents one individual. **H** Western blot analysis of PmCQ2-infected TC-1 cells with or without shFoxo1 knockdown at 6 hpi. **I** Flow cytometry analysis of apoptosis in PmCQ2-infected TC-1 cells with or without shFoxo1 knockdown at 6 hpi. Each group contained 3 replicates. The data are presented as the means ± SDs. *p < 0.05, **p < 0.01, ***p < 0.001, N. S: not significant.

**Figure 6:**
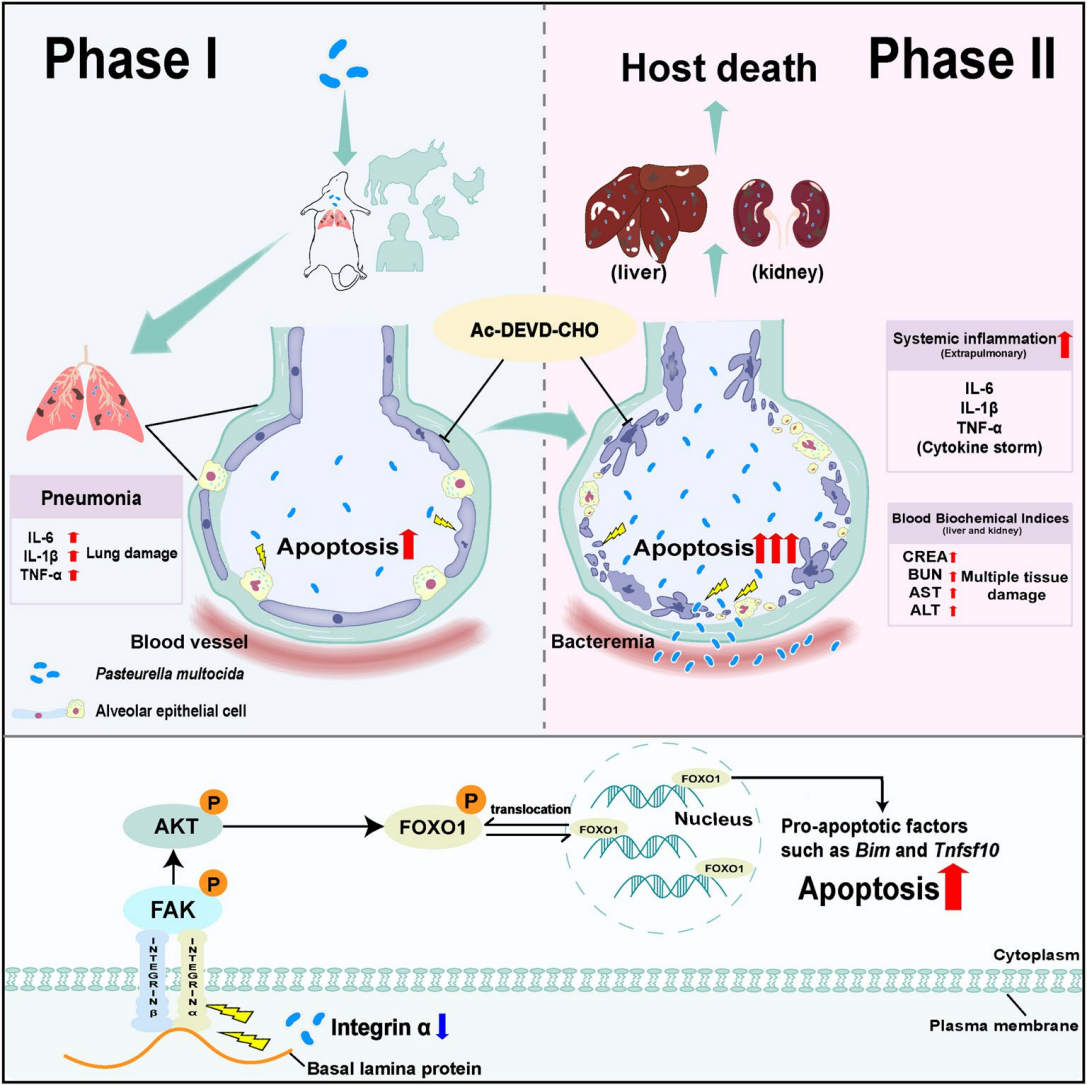
**Schematic showing that *Pasteurella multocida *activates apoptosis via the FAK-AKT-FOXO1 axis to cause pulmonary integrity loss, bacteremia, and eventually a cytokine storm**. The complete model of *P. multocida* infection consists of at least two phases: the pulmonary infection phase (Phase 1) and the systemic infection phase (Phase 2). At the molecular level, *P. multocida* inhibits the FAK-AKT-FOXO1 axis by regulating integrin alpha family protein expression to induce excessive pulmonary epithelial cell apoptosis, eventually resulting in systemic infection. In contrast, targeting apoptosis with particular inhibitors can attenuate *P. multocida* infection.

To further explore the exact molecular mechanism of *P. multocida*-induced excessive apoptosis in the lungs, we performed RNA-seq again at the onset of bacteremia (16 hpi). By comparing the two RNA-seq datasets, we found that focal adhesion and its downstream AKT pathway can be involved in *P. multocida*-induced apoptosis (Figure 5A; Additional file 6A). According to the most recent RNA-seq data, the mRNA levels of most integrin α family proteins were decreased, which was subsequently confirmed by qPCR (Figure 5B; Additional file 6B). Integrins are the key receptor proteins that are heterodimers composed of α subunits and β subunits. Integrin α/β heterodimeric cell surface receptors play a pivotal role in cell adhesion and migration, as well as in growth and survival [33, 34]. Integrin αV is expressed in various tissues and cell types, including endothelial cells, epithelial cells and fibroblasts, and αVβ3, αVβ5, and αVβ1 regulate epithelial cell proliferation and survival [35]. Furthermore, we confirmed that the expression of integrin αV and the downstream kinase p-Fak was obviously downregulated in vitro and in vivo (Figures 5C and D; Additional file 7A), suggesting that *P. multocida* inhibited focal adhesion. Given that the antiapoptotic factor AKT is the downstream factor of focal adhesion, we found that p-AKT was correspondingly downregulated in the mouse models and rabbit models (Figure 5E; Additional file 7B). In addition, we found that a series of FOXO1-induced genes, including apoptotic genes, were upregulated after *P. multocida* infection (Figure 5F), suggesting that *P. multocida* can activate FOXO1 through the suppression of AKT. We found that lethal PmCQ2 disrupted the FAK-AKT-FOXO1 axis and increased the levels of the apoptosis executor Cleaved Caspase-3 both in vitro and in vivo (Figure 5G; Additional file 7C). Conversely, low-virulence PmCQ6 failed to disrupt the FAK-AKT-FOXO1 pathway, indicating that disruption of the FAK-AKT-FOXO1 axis may be associated with *P. multocida*-induced host death. Next, we tested whether FOXO1 indeed contributed to *P. multocida*-induced apoptosis. As shown in Additional file 7D, knockdown of FOXO1 using shRNA significantly reduced FOXO1 expression in TC-1 cells. Then, we measured apoptosis in TC-1 cells infected with PmCQ2 and found that the expression of *P. multocida*-induced Cleaved Caspase-3 and the rate of apoptosis were significantly lower in TC-1 cells after knockdown of FOXO1 than in those infected with shGFP (Figs. 5H and I), indicating that FOXO1 indeed contributes to *P. multocida*-induced pulmonary epithelial cell apoptosis. Taken together, the abovementioned findings indicated that *P. multocida* disrupts the pulmonary epithelial FAK-AKT-FOXO1 axis to induce apoptosis. A hypothetical figure is provided in Figure 6.

## Discussion

*Pasteurella multocida*, an opportunistic pathogen, is common in the upper respiratory tract of a variety of animals, such as cattle, goats, and rabbits, and is responsible for human respiratory diseases [36–38]. Notably, although *P. multocida* infection manifests as respiratory symptoms, extrapulmonary infections, including infections of the liver, kidney, heart, spleen and gut, are also common in the clinic, indicating that *P. multocida* not only damages the respiratory tract but also causes systemic infection [39–41]. Therefore, in the present study, we demonstrated that *P. multocida* not only causes severe lung damage but also induces systemic infection via bacteremia. Importantly, to our knowledge, we are the first to show that bacteremia is an essential step in *P. multocida* pathogenesis because it greatly accelerates host death.

The phenomenon known as the “cytokine storm” has gained notoriety since the respiratory pathogen COVID-19 causes damage to the lungs and several extrapulmonary organs and triggers the excessive release of inflammatory cytokines [42]. The cytokine storm is a life-threatening systemic inflammatory syndrome characterized by multiple organ damage and excessive release of inflammatory cytokines, particularly IL-6, TNF-α and IL-1β [31]. Cytokine storms have been shown to contribute to the pathogenesis of certain bacteria, such as *Yersinia pestis* and *Salmonella enteritidis* [43, 44]. In the present study, we found that *P. multocida* infection indeed damaged multiple organs and induced systemic inflammation. Importantly, anti-IL6 neutralizing antibodies, most commonly used to treat cytokine storms [45, 46], had a significant protective effect against *P. multocida* infection, providing the first evidence that *P. multocida* can cause a cytokine storm. Based on these findings, we propose that complete *P. multocida* infection consists of at least two stages, namely, the pulmonary infection phase (phase 1) and the systemic infection phase (phase 2). Our present results also remind researchers who study respiratory pathogens, including ourselves, to review the results obtained with nonrespiratory infection models, such as the intraperitoneal injection model [25, 47], which ignores phase 1 and jumps directly to phase 2.

We aimed to understand how *P. multocida* breaks through the pulmonary epithelial-blood barrier. In the present study, our RNA-seq results showed that the apoptosis pathway may be involved in the loss of the pulmonary epithelial-blood barrier. This result is also consistent with our previous transcriptome analysis showing that apoptosis was one of the top ten pathways enriched in *P. multocida*-infected mouse lungs [26]. The abnormal occurrence of apoptosis, especially excessive apoptosis, contributes to the loss of physiological barriers, such as the respiratory barrier and gut barrier [48, 49]. Bacterial pathogens, such as *Listeria monocytogenes* and *Staphylococcus aureus*, can manipulate apoptosis to promote its pathogenesis [50, 51]. Here, we found that *P. multocida* induced pulmonary epithelial cell apoptosis, which contributed to pulmonary integrity loss. Importantly, treatment with the apoptosis inhibitor Ac-DEVD-CHO significantly reduced *P. multocida*-induced murine death, suggesting that pulmonary epithelial cell apoptosis contributes to *P. multocida* pathogenesis. To our knowledge, this is the first time that a role for apoptosis in *P. multocida* infection has been identified.

To identify important pathways and understand the underlying mechanism of *P. multocida*-induced apoptosis, a new RNA-seq study at the onset of bacteremia was performed. Moreover, compared with previous RNA-seq data, both RNA-seq data revealed that apoptosis-associated pathways, especially the focal adhesion and AKT pathways, were enriched. Notably, focal adhesion can directly influence AKT activity via FAK [52], while AKT can directly regulate apoptosis by influencing downstream effectors, such as FOXO1 and P53 [53, 54]. Focal adhesion is an important connection between cells and the external matrix, and the formation and stability of focal adhesions play important roles in cell apoptosis, proliferation, and other processes [35]. Focal adhesions are composed of several proteins, among which integrins, dimers composed of an alpha subunit and a beta subunit, are the main cell adhesion receptors [35]. A recent study also suggested that the key virulence protein of *P. multocida*, OmpA, has an impact on focal adhesion [55]. In the present study, we found that the expression of integrin α proteins and the downstream effector p-Fak decreased in vivo after *P. multocida* infection, suggesting that *P. multocida* inhibited focal adhesion. Accordingly, the phosphorylation of AKT and its downstream effectors p-mTOR and p-FOXO1 are also markedly reduced. FOXO1 manipulates apoptosis via the transcription of apoptosis-associated genes such as *Bim* and *Tnfsf10*. In contrast, the knockdown of FOXO1 significantly inhibited *P. multocida*-induced apoptosis, suggesting that *P. multocida* promotes pulmonary epithelial cell apoptosis by inhibiting the FAK-AKT-FOXO1 pathway. Considering that *P. multocida* mainly infects domestic animals, we validated the key results from the mouse model in a rabbit model. Additionally, a recent study revealed that the HIF-1α–VEGFA axis contributes to *P. multocida*-induced epithelial permeability [56], but our RNA-seq data revealed that apoptosis and the AKT pathway are more important than HIF-1α.

In conclusion, our current work indicated that a complete model of *P. multocida* infection consists of at least two phases: the pulmonary infection phase (Phase 1) and the systemic infection phase (Phase 2). Furthermore, we demonstrated that *P. multocida*-induced pulmonary epithelial cell apoptosis is a crucial part of *P. multocida* pathogenesis and contributes to pulmonary integrity loss, bacteremia and cytokine storms. The underlying mechanism by which *P. multocida* induces apoptosis is associated with dysregulation of the FAK-AKT-FOXO1 pathway. Importantly, we confirmed that the inhibition of pulmonary epithelial cell apoptosis by a selective inhibitor can attenuate bacterial respiratory pathogen infection in mouse models, revealing these two novel targets as potential therapeutic strategies against *P. multocida* and other respiratory bacterial infections (Figure 6).

### Supplementary Information

**Additional file 1: Original RNA-seq data used in this study**.

**Additional file 2: Quantification of IL-6, TNF-α, and IL-1β in the lungs of mice infected with or without PmCQ2 by ELISA at 16 hpi**.

**Additional file 3: HE staining of rabbit lungs intranasally infected with 10**^**8**^** CFU of PmCQ2 at 24 hpi and blood bacterial load of rabbits intranasally infected with 10**^**8**^** CFU of PmCQ2 at 24 hpi**.

**Additional file 4: TUNEL staining of rabbit livers intranasally infected with 10**^**8**^** CFU of PmCQ2 at 24 hpi**.

**Additional file 5: TUNEL staining of rabbit lungs intranasally infected with 10**^**8**^** CFU of PmCQ2 at 24 hpi**.

**Additional file 6: Bioinformatics analysis based on RNA-seq**. **A** Top 15 enriched signalling pathways according to RNA-seq of mouse lungs infected with PmCQ2 at 40 hpi. **B** KEGG enrichment pathway of PI3K-AKT. On the right is the heatmap of integrin family protein expression according to RNA-seq at 16 hpi. The red box indicates the focal adhesion pathway.

**Additional file 7: WB analysis of the Fak-Akt-Foxo1 axis in TC-1 cells and p-AKT expression in rabbit lungs**. **A** Western blot analysis of integrin αV, p-Fak and Fak in TC-1 cells infected with PmCQ2 at an MOI of 1 for 6 h. **B** IHC staining of p-Akt in rabbit lungs intranasally infected with 10^8^ CFU of PmCQ2 at 24 hpi. **C** Western blot analysis of the AKT-FOXO1 pathway and cleaved caspase 3 as well as 7 in TC-1 cells infected with 1 MOI PmCQ2 for 6 h. **D** Western blot analysis of Foxo1 expression in TC-1 cells treated with or without shFoxo1.

**Additional file 8: qPCR sequences of primers used in this study**.

## Data Availability

The data used to support the findings of this study are available from the corresponding author upon reasonable request.
