# Supplementary figures and images for: Pasteurella multocida activates apoptosis via the FAK-AKT-FOXO1 axis to cause pulmonary integrity loss, bacteremia, and eventually a cytokine storm
Source: Vet Res. 2024 Apr 8;55:46. doi: 10.1186/s13567-024-01298-7 (PMC11003142; doi:10.1186/s13567-024-01298-7)

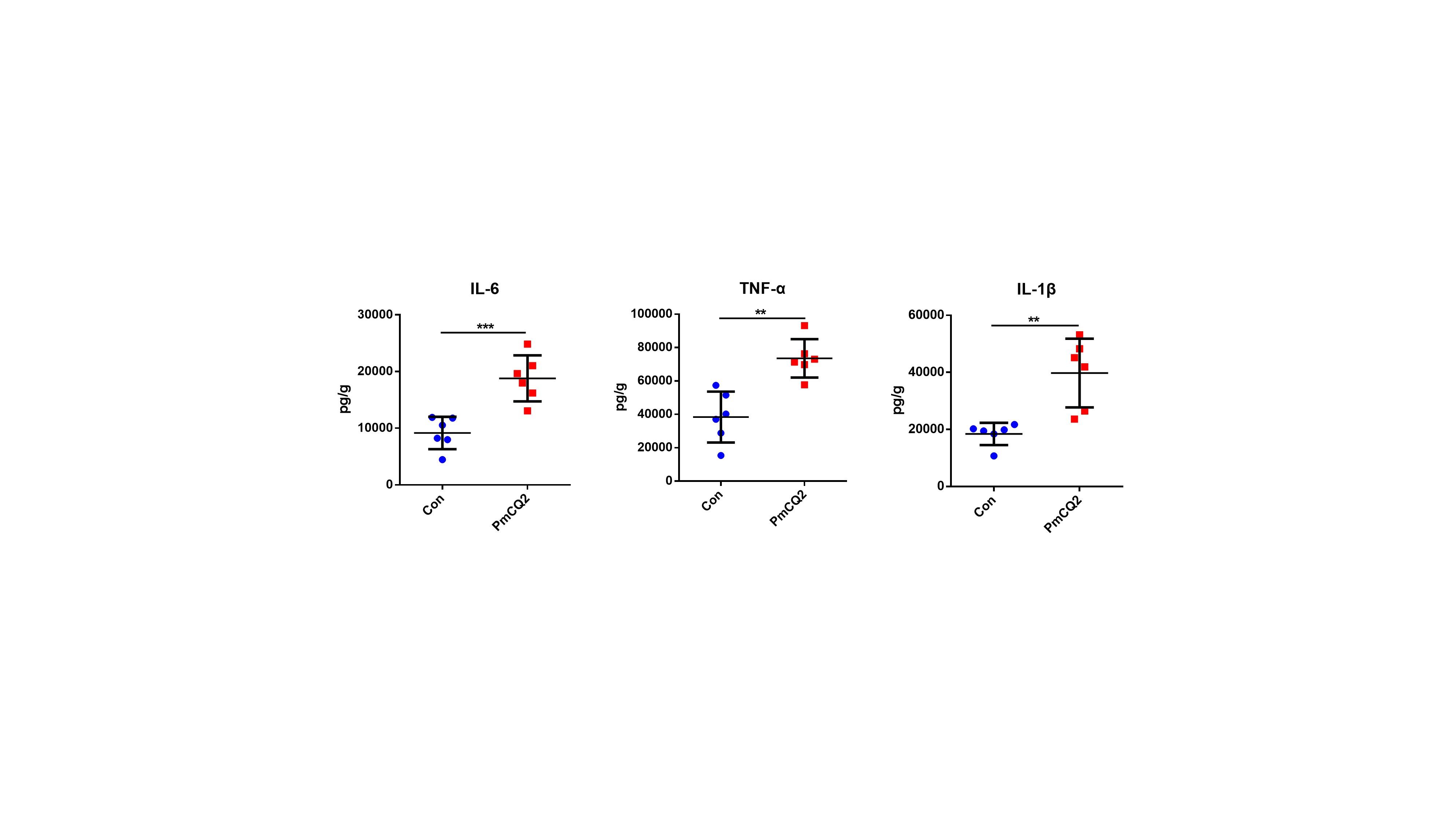

Supplement: Supplementary file 2 — Additional file 2: Quantification of IL-6, TNF-α, and IL-1β in the lungs of mice infected with or without PmCQ2 by ELISA at 16 hpi. [file 13567_2024_1298_MOESM2_ESM.jpg]

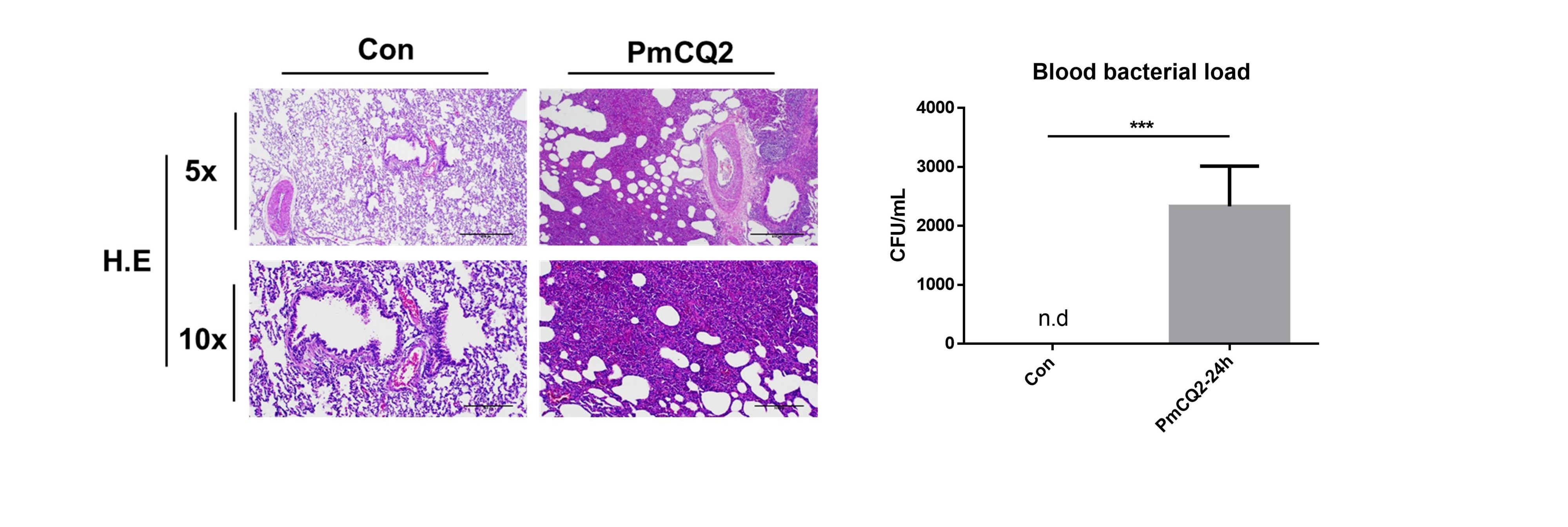

Supplement: Supplementary file 3 — Additional file 3: HE staining of rabbit lungs intranasally infected with 108 CFU of PmCQ2 at 24 hpi and blood bacterial load of rabbits intranasally infected with 108 CFU of PmCQ2 at 24 hpi. [file 13567_2024_1298_MOESM3_ESM.jpg]

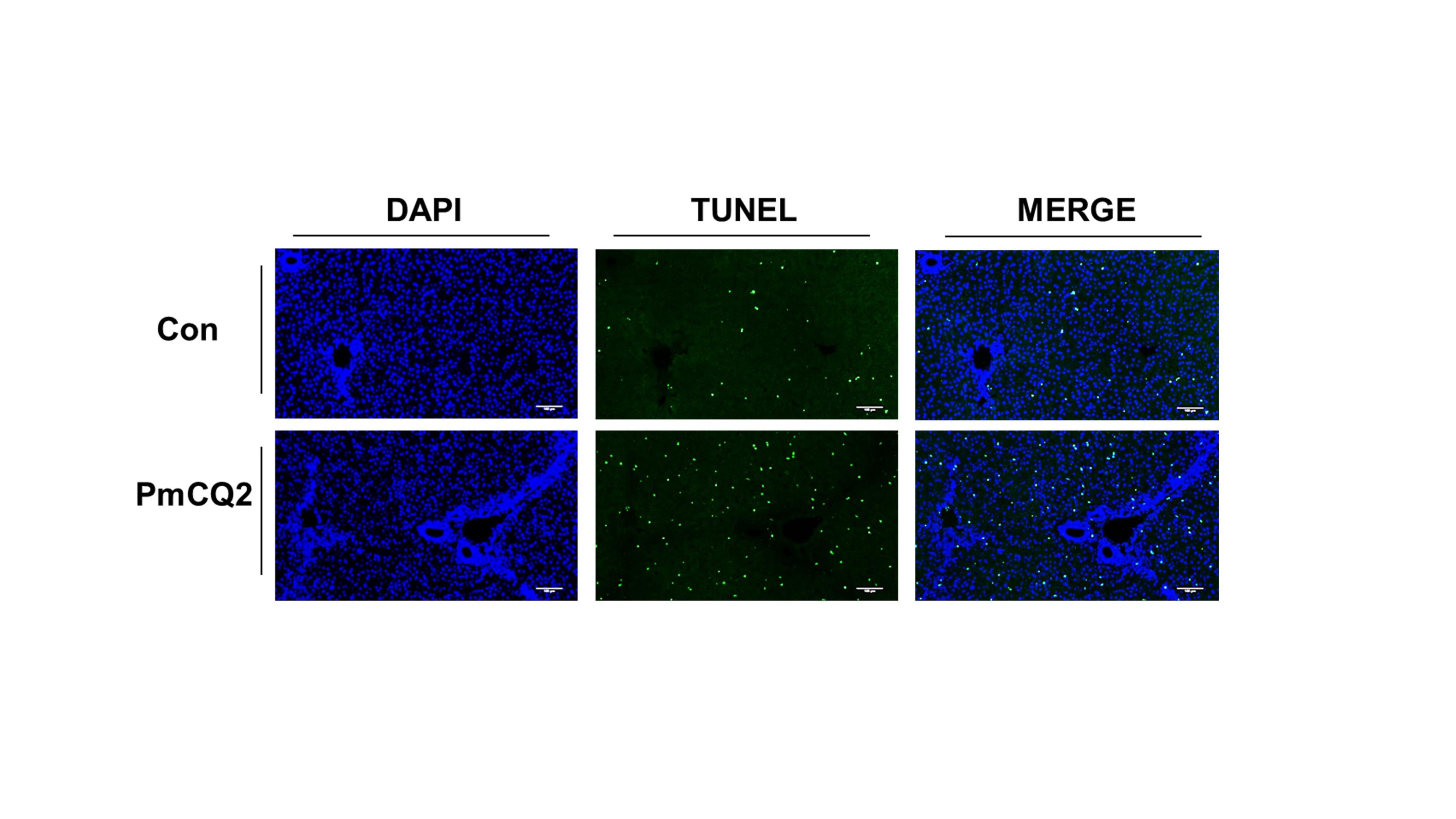

Supplement: Supplementary file 4 — Additional file 4: TUNEL staining of rabbit livers intranasally infected with 108 CFU of PmCQ2 at 24 hpi. [file 13567_2024_1298_MOESM4_ESM.jpg]

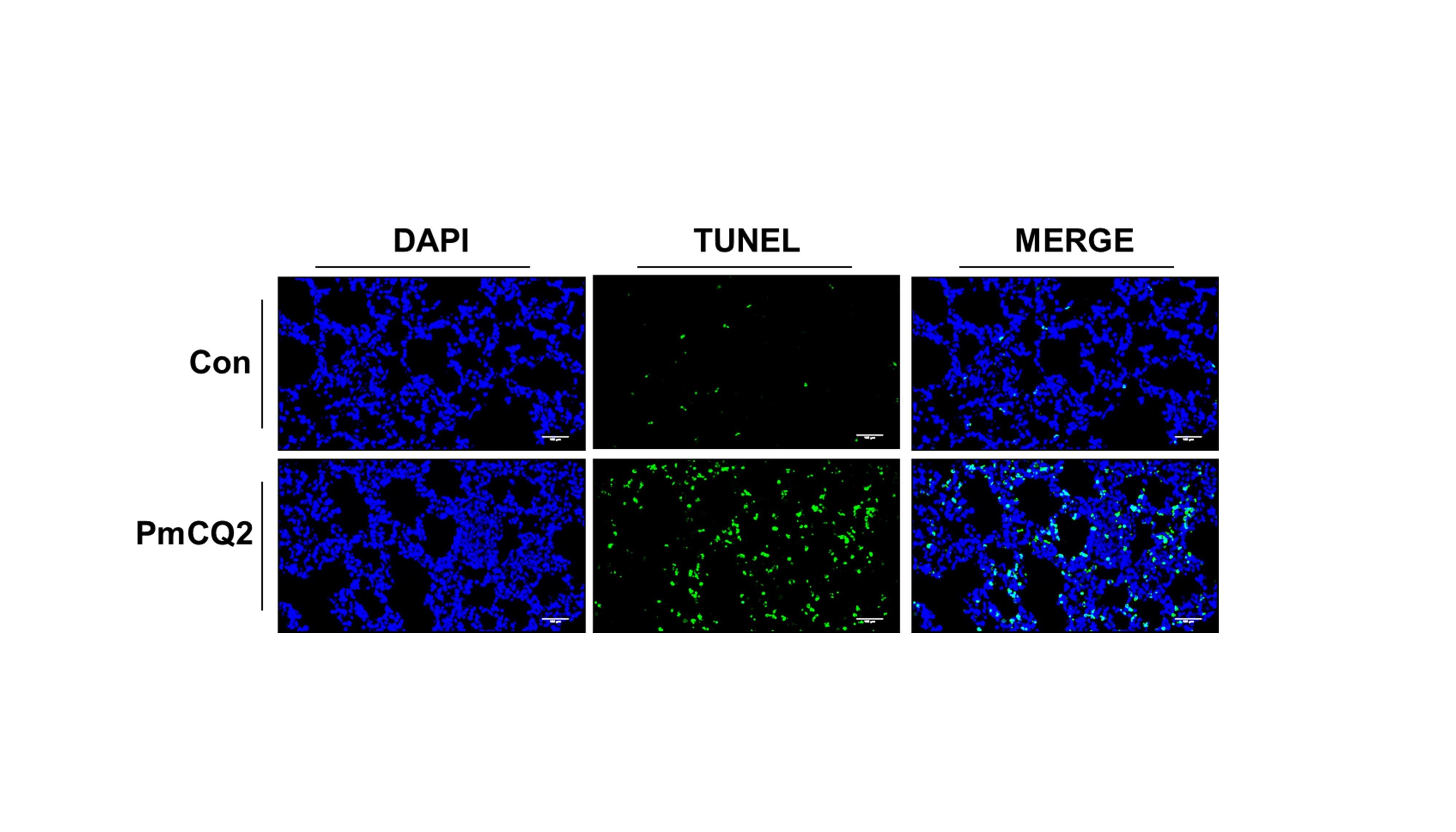

Supplement: Supplementary file 5 — Additional file 5: TUNEL staining of rabbit lungs intranasally infected with 108 CFU of PmCQ2 at 24 hpi. [file 13567_2024_1298_MOESM5_ESM.jpg]

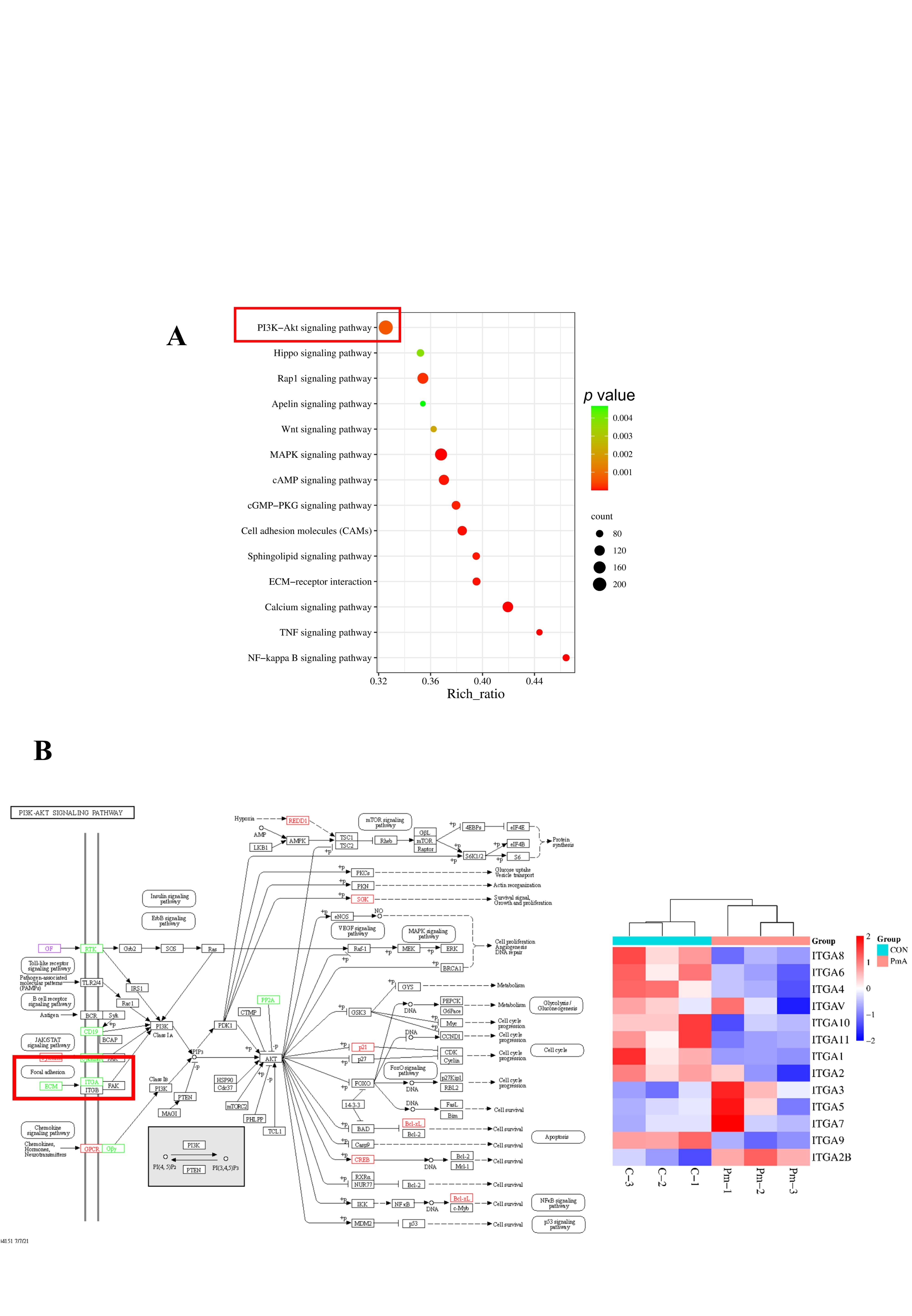

Supplement: Supplementary file 6 — Additional file 6: Bioinformatics analysis based on RNA-seq. A Top 15 enriched signalling pathways according to RNA-seq of mouse lungs infected with PmCQ2 at 40 hpi. B KEGG enrichment pathway of PI3K-AKT. On the right is the heatmap of integrin family protein expression according to RNA-seq at 16 hpi. The red box indicates the focal adhesion pathway. [file 13567_2024_1298_MOESM6_ESM.jpg]

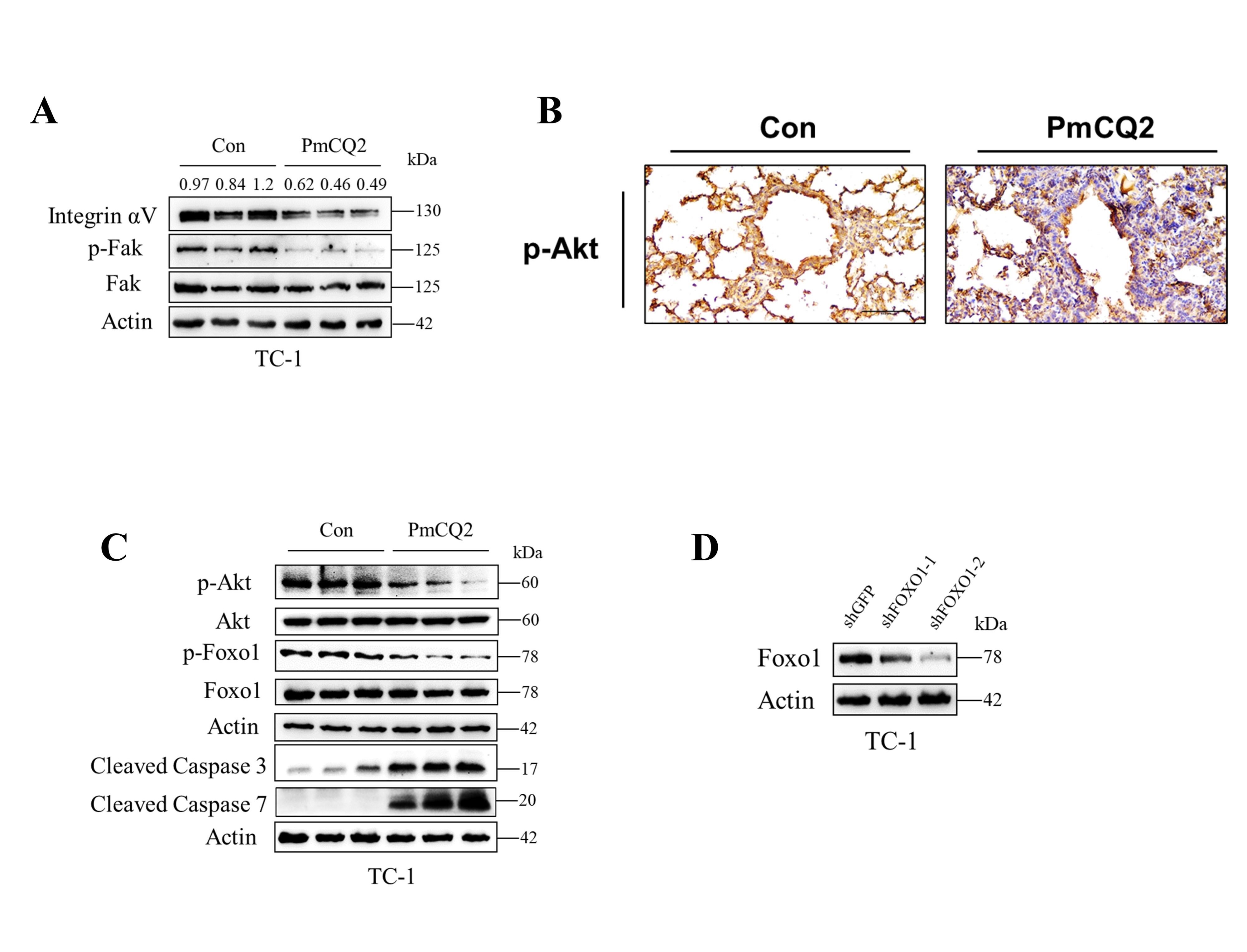

Supplement: Supplementary file 7 — Additional file 7: WB analysis of the Fak-Akt-Foxo1 axis in TC-1 cells and p-AKT expression in rabbit lungs. A Western blot analysis of integrin αV, p-Fak and Fak in TC-1 cells infected with PmCQ2 at an MOI of 1 for 6 h. B IHC staining of p-Akt in rabbit lungs intranasally infected with 108 CFU of PmCQ2 at 24 hpi. C Western blot analysis of the AKT-FOXO1 pathway and cleaved caspase 3 as well as 7 in TC-1 cells infected with 1 MOI PmCQ2 for 6 h. D Western blot analysis of Foxo1 expression in TC-1 cells treated with or without shFoxo1. [file 13567_2024_1298_MOESM7_ESM.jpg]
