# Supplementary material for: Pasteurella multocida activates apoptosis via the FAK-AKT-FOXO1 axis to cause pulmonary integrity loss, bacteremia, and eventually a cytokine storm
Source: Vet Res. 2024 Apr 8;55:46. doi: 10.1186/s13567-024-01298-7 (PMC11003142; doi:10.1186/s13567-024-01298-7)
Supplement: Supplementary file 8 — Additional file 8: qPCR sequences of primers used in this study. [file 13567_2024_1298_MOESM8_ESM.pdf]

Gene	gene ID		Sequence
ITGA8	241226	ITGA8-F	TGGCTGGGATTCCAAGAGGA
		ITGA8-R	GTGCCCCGACCAATATGTCA
ITGA6	16403	ITGA6-F	TGCAGAGGGCGAACAGAAC
		ITGA6-R	GCACACGTCACCACTTTGC
ITGA4	16401	ITGA4-F	GATGCTGTTGTTGTACTTCGGG
		ITGA4-R	ACCACTGAGGCATTAGAGAGC
ITGAV	16410	ITGAV-F	CCGTGGACTTCTTCGAGCC
		ITGAV-R	CTGTTGAATCAAACTCAATGGGC
ITGA10	213119	ITGA10-F	TCCGGTCTTCCAGACTGGG
		ITGA10-R	GAGCTGTGTTATCTTGAAGGGTC
ITGA11	319480	ITGA11-F	TGCCCCAATGGAAACCAATG
		ITGA11-R	CACTCGTGCGACCAGAGAG
ITGA1	109700	ITGA1-F	CCTTCCCTCGGATGTGAGTCA
		ITGA1-R	AAGTTCTCCCCGTATGGTAAGA
ITGA2	16398	ITGA2-F	TGTCTGGCGTATAATGTTGGC
		ITGA2-R	CTTGTGGGTTCGTAAGCTGCT
ITGA3	16400	ITGA3-F	CCTCTTCGGCTACTCGGTC
		ITGA3-R	CCGGTTGGTATAGTCATCACCC
ITGA5	16402	ITGA5-F	CTTCTCCGTGGAGTTTTACCG
		ITGA5-R	GCTGTCAAATTGAATGGTGGTG
ITGA7	16404	ITGA7-F	CTGCTGTGGAAGCTGGGATTC
		ITGA7-R	CTCCTCCTTGAACTGCTGTCG
ITGA9	104099	ITGA9-F	AAGTGTCGTGTCCATACCAAC
		ITGA9-R	GGTCTGCTTCGTAGTAGATGTTC
ITGA2B	16399	ITGA2B-F	TTCTTGGGTCCTAGTGCTGTT
		ITGA2B-R	CGCTTCCATGTTTGTCCTTATGA
Tnfsf10	22035	Tnfsf10-F	ATGGTGATTTGCATAGTGCTCC
		Tnfsf10-R	GCAAGCAGGGTCTGTTCAAGA
Bim	12125	Bim-F	GACAGAACCGCAAGACAGGAG
		Bim-R	GGACTTGGGGTTTGTGTTGAC
Plk3	12795	Plk3-F	GCACATCCATCGGTCATCCAG
		Plk3-R	GCCACAGTCAAACCTTCTTCAA
Klf-2	16598	Klf-2-F	CTCAGCGAGCCTATCTTGCC
		Klf-2-R	CACGTTGTTTAGGTCCTCATCC
β-Actin	11461	β-Actin-F	GGCTGTATTCCCCTCCATCG
		β-Actin-R	CCAGTTGGTAACAATGCCATGT
